# Recent Updates in Redox Regulation and Free Radical Scavenging Effects by Herbal Products in Experimental Models of Parkinson’s Disease

**DOI:** 10.3390/molecules171011391

**Published:** 2012-09-26

**Authors:** Sushruta Koppula, Hemant Kumar, Sandeep Vasant More, Hyung-Woo Lim, Soon-Min Hong, Dong-Kug Choi

**Affiliations:** Department of Biotechnology, Konkuk University, Chungju 380-701, Korea

**Keywords:** Parkinson’s disease, oxidative stress, redox signaling, antioxidant herbs, reactive oxygen species, 6-hydroxydopamine, substantia nigra

## Abstract

Parkinson’s disease (PD) is a complex multifactorial disease marked by extensive neuropathology in the brain with selective yet prominent and progressive loss of mid-brain dopaminergic neurons. The etiological factors involved in the development of PD are still elusive, but oxidative stress arising when reactive oxygen species (ROS) exceed amounts required for normal redox signaling is considered one of the major factors. ROS cause oxidative damage to proteins, lipids, and DNA and are one of the most prominent factors related to neurodegeneration. Pre-clinical and clinical studies clearly demonstrate the effectiveness of oxidative stress in the pathogenesis of PD. Therefore, regulation of redox signaling and inhibiting excess ROS would contribute greatly not only to extend longevity but also to ameliorate the progression of dopaminergic cell death seen in patients with PD. Several herbal products are beneficial for maintaining nerve cell function and for treating various neurodegenerative disorders by reducing oxidative stress. Here, we summarize the recent knowledge concerning promising herbs that have shown significant beneficial effects based on regulation of redox status and ROS inhibition in toxin-induced PD models.

## 1. Introduction

Parkinson’s disease (PD) is the second most common neurodegenerative disorder of the central nervous system after Alzheimer’s disease (AD). It has been estimated that 1% of individuals >65 years are diagnosed with this disorder, and that this figure may double by the year 2030. PD might be a serious socioeconomic burden in a future aging society [1,2,3]. PD particularly affects a region of the basal ganglia called the *substantia nigra* (SN), which produces dopamine (DA). DA acts as a messenger between the SN and another area of the brain called the *corpus striatum*. Together, they coordinate smooth flowing muscle movement. Inadequate DA levels result in specific neurological symptoms such as tremors, difficulty walking, rigidity, slowed movements, and a decreased ability to make spontaneous movements, which characterize PD. In advanced stages, mental impairment has also been observed. There is a general agreement that PD may represent the final outcome of interactions among multiple factors, including genetic susceptibility and exposure to environmental toxins [4,5,6].

A great deal has been learned about PD, yet a cure has not been found. Current initial therapy includes DA replacement strategies with L-dopa, dopaminergic agonists, monoamine oxidase B inhibitors, and drug combinations that provide dramatic improvement and have become the accepted way to control PD symptoms. Other medications include catechol-*o*-methyltransferase, anticholinergic agents, and amantadine [7], but one of the problems when using these therapies is that they lose effectiveness over time. Other problems are the side effects of nausea, vomiting, headache, fatigue, fainting, increased thirst, and tremors. Thus, new approaches for handling PD are clearly needed.

## 2. Redox Regulation of Neuronal Survival in PD

Research disciplines focusing on oxidative stress have increased our knowledge of importance of the cell’s redox status and the recognition of oxidative stress as a process with implications for many pathophysiological states. Oxidative stress may be defined as an imbalance between oxidant-producing systems and antioxidant mechanisms (redox balance), which results in excess formation of reactive oxygen species (ROS) [8]. The delicate balance between ROS generation and elimination is maintained by many complex mechanisms, and a dysfunction in any of these mechanisms can lead to alterations in cellular redox status. An increase in ROS production or a decrease in ROS-scavenging capacity due to exogenous stimuli or endogenous metabolic alterations can disrupt redox homeostasis, leading to an overall increase in intracellular ROS levels or oxidative stress [9]. Previous studies have suggested that the regulation of signaling pathways by the redox system relies mostly on direct oxidative modifications of redox-sensitive signaling proteins. Redox regulation of neuronal survival at the transcription level, which includes alterations in transcription factors such as nuclear factor-κB (NF-κB), activating protein-1 (AP-1), nuclear factor erythroid 2 (Nrf2), and hypoxia inducible factor (HIF) may affect overall oxidative status [10]. Furthermore, redox regulation of neuronal survival at the signal-transduction level influences the mitogen-activated protein kinase and PI3K/Akt pathways, as well as several redox sensitive apoptotic effectors such as caspases, Bcl-2, and cytochrome *c*, and their functions can be significantly affected by cellular ROS [11,12,13,14,15]. The consequence of oxidative stress leading to neuronal survival or death is likely dependent on the integration of these redox-sensitive signals. 

Oxidative stress has been consistently linked to ageing related diseases and has emerged as a causative factor in the pathology of several neurodegenerative disorders including PD, AD, and amyotrophic lateral sclerosis. Neurodegenerative diseases are characterized by progressive dysfunction and death of neurons. A steady-state balance exists between pro-oxidants and antioxidants under normal conditions. However, when the rate of free radical generation exceeds the capacity of antioxidant defenses, oxidative stress ensues with consequential severe damage to cellular machinery. Oxidative stress is associated with mitochondrial and endoplasmic reticulum dysfunction, inducing apoptosis and protein misfolding in neurons. Decreased activities of antioxidant enzymes such as superoxide dismutase (SOD), catalase (CAT), glutathione (GPx), and glutathione peroxidase in neurodegenerative states signify the role of reduced antioxidant potential during neurodegeneration. Oxidative stress results from a number of endogenous sources, including the production of oxygen-free radicals by mitochondrial oxidative phosphorylation, which is the primary source of high-energy compounds in the cell. Mitochondria are a significant source of ROS, as they are the major consumers of molecular oxygen in cells [16,17]. Mitochondrial dysfunction leads to reduced ATP production, increased oxidative stress, altered mitochondrial morphology, impaired calcium buffering, damage to mitochondrial DNA, and alterations in mitochondrial fission and fusion, eventually leading to cell death [18]. Mitochondrial complex I alterations are a major source of ROS generation in patients with PD. An inhibited mitochondrial complex hampers the mitochondrial respiratory chain, which causes incomplete oxygen reduction, thereby generating reactive species including deleterious O_2_^−^, which is further converted to NO_3_^−^ and, finally, to ˙OH through the Fenton reaction [19]. Thus, dysfunctional mitochondria are the primary intracellular ROS source contributing to oxidative stress-mediated neurodegeneration in PD models [20,21,22].

## 3. Free Radicals Scavengers and PD

Free radicals are generated continuously via normal physiological events such as respiration and some cell-mediated immune functions. ROS molecules are simply oxygen-containing molecules that are highly reactive. They can be divided into free-radical ROS, which have unpaired electrons in their outer orbits (e.g., superoxide and hydroxyl radicals) and nonradical ROS, which lack unpaired electrons, but are chemically reactive and can be converted to free-radical ROS (e.g., hydrogen peroxide). The generation of large amounts of these species results in attacks on intracellular antioxidant defense causing activation of lipid peroxidation, protein modification, and oxidative DNA damage [23,24,25,26,27]. These reactive species and oxidative stress are particularly involved in neurodegenerative diseases, as the brain uses 20% of the inspired oxygen and 90% of the consumed oxygen to produce energy during oxidative phosphorylation. Neuronal cells in the brain are particularly sensitive and vulnerable to oxidative damage because of their high metabolic activity, low antioxidant capacity, and non-replicative nature [28]. 

The idea that excessive numbers of free radicals play an important role in the complex etiology of PD has spread in the last few years. *Post-mortem* investigations have revealed evidence of lipid peroxidation and oxidative damage to brain protein DNA in patients suffering from PD [29]. The increase in oxidative stress is attributed to a less active mitochondrial complex I among other factors. The role of free radicals and oxidative stress in PD has been extensively studied, and the results obtained make a compelling argument for the concept that oxidative stress occurs in the brains of patients with PD [30,31,32,33,34]. To a large extent, animal models of PD, in which the neurotoxins 1-methyl-4-phenyl-1,2,3,6-tetrahydropyridine (MPTP) and 6-hydroxydopamine (6-OHDA) are administered, confirm these findings [35,36]. Furthermore, *substantia nigra* samples from PD brains also have reduced levels of antioxidants and antioxidant enzymes [27,37,38,39,40]. These changes increase the balance in favor of a pro-oxidant environment, which increases oxidative damage. Because ROS accumulation promotes excess or premature neuronal death leading to neurodegenerative diseases, the use of free radical scavengers or antioxidants to prevent neurodegeneration has been established for decades. This treatment involves the use of both supplementations with natural ROS scavengers as well as treatment with exogenous antioxidants. Several radical scavenger antioxidants show neuroprotective effects in toxin-induced PD models. A proposition that is widely accepted is that supplementing antioxidants reduces the risk of suffering from PD or delays its progression [41]. Molecules such as cysteine, melatonin, resveratrol, and ebselen are well demonstrated in experimental models to be neuroprotective based on their antioxidant properties [42,43,44,45]. Additionally, regular consumption of diets rich in antioxidants such as those found in fruits and vegetables may reduce the risk of developing neurodegenerative diseases such as PD and AD [46]. Antioxidants are capable of transforming ROS into stable and harmless compounds or by scavenging based on redox mechanism. Antioxidants keep our cells clean by combining with and eliminating very dangerous reactive molecules that can cause random changes in proteins, resulting in degeneration of dopaminergic (DAergic) neurons seen in PD [47,48].

## 4. Oxidative Stress and Toxin-Induced PD Models

Intracellular redox homeostasis is disturbed by oxidative stress, which manifests itself through dysregulation in the balance between systems that produce oxidant agents and the antioxidant defense mechanisms (the redox balance). Experimental models to produce prototypic oxidative stress and selective neuronal death and induction with selected neurotoxins have been utilized to investigate this mechanism. Neurotoxins have remained the most popular tools to obtain greater insights into oxidative stress and redox signaling mechanisms. Among the neurotoxins used to induce DAergic neurodegeneration, 6-OHDA, MPTP, and, more recently, paraquat and rotenone have received the most attention. Presumably, all of these toxins provoke ROS formation. Rotenone and MPTP are similar in their ability to potently inhibit mitochondrial complex I, although they display significant differences, including their ease of use in animals. 

6-OHDA enters both DAergic and noradrenergic neurons and inflicts damage to the catecholaminergic pathways in both the peripheral and central nervous systems. 6-hydroxydopamine, the first animal model of PD associated with *substantia nigra pars compacta* (SNpc) DAergic neuronal death was introduced more than 30 years ago [49]. It is well accepted that 6-OHDA destroys catecholaminergic structures thorough a combined effect on ROS and quinones [50]. Once dissolved in an aerobic and alkaline milieu, 6-OHDA readily oxidizes yielding hydrogen peroxide and *p*-quinone. Further information regarding 6-OHDA is reviewed in Przedborski and Ischiropoulos [20]. 

The human Parkinsonian neurotoxin MPTP has been used in a variety of animals to model PD from nonhuman primates to invertebrates such as worms [51,52]. Neuropathological data in both humans and monkeys indicate that MPTP causes damage to the nigrostriatal DAergic pathway identical to that seen in PD [53,54]. MPTP causes DAergic neurodegeneration by generating free radicals and leading to oxidative stress as shown by alterations in the states of antioxidant enzymes and molecules [55,56,57]. MPTP per se is nontoxic to neurons, but the enzyme monoamine oxidase B converts MPTP to 1-methyl-4-phenylpyridinium (MPP^+^) in the brain, which enters DA terminals via DA uptake sites. Within the DA terminals, MPP^+^ blocks mitochondrial complex I and causes ATP depletion. This is thought to be the main cause of MPTP-induced terminal degeneration. Additionally, MPP^+^ produces oxidative stress by generating ROS after blocking mitochondrial complex I and DA oxidation, which may participate in MPTP-induced DAergic toxicity [58,59,60].

Epidemiological studies suggest that exposure to pesticides may be a risk factor for PD. One of the most important pesticides used widely around the world is rotenone. Due to its toxic effects, which resemble PD, natural rotenone represents one of the most recently used approaches in toxic animal models of PD. Similar to MPTP, rotenone is highly lipophilic and readily gains access to all organs including the brain. Rotenone freely crosses all cellular membranes in the brain and accumulates in subcellular organelles such as mitochondria to inhibit mitochondrial complex I. This inhibition has several potentially damaging consequences, including increased formation of ROS, which cause oxidative damage within the cell. Oxidative damage, rather than a bioenergetic defect, is also seen in an *in vivo* rotenone model [61,62].

The potent herbicide paraquat (*N*,*N*-dimethyl-4-4-bipiridinium) is another prototypic toxin that exerts deleterious effects through oxidative stress. Paraquat toxicity appears to be mediated by the formation of superoxide radicals [63]. Furthermore, paraquat toxicity is also mediated by redox cycling with cellular diaphorase such as nitric oxide synthase yielding ROS [63]. Paraquat exhibits a striking structural similarity to the MPTP toxic metabolite MPP^+^. The actual reduction-oxidation cycling reaction of paraquat has been reviewed by Przedborski and Ischiropoulos [20]. 

Along with the four most popular toxic models of PD discussed above, several other toxins such as manganese (Mn), kainite, methamphetamine, and isoquinoline are believed to damage DAergic neurons based on oxidative stress mechanisms by producing ROS and disturbing overall redox status [20]. However, each model has its own advantages and shortcomings. Several natural antioxidant products act as neuroprotective agents in PD models by targeting oxidative stress and redox regulation mechanisms. Over 4,000 species of plant-derived antioxidants have protective effects against the neurodegeneration induced by elevated ROS levels [64]. Active constituents such as green tea polyphenols, resveratrol, uric acid, curcumin, quercetin, CoQ10, creatinine and several traditional herbal medicines have proven protective against toxin-induced models of PD [65,66,67,68,69,70]. The use of several neurotoxins as experimental models to reproduce many of the clinical hallmarks of PD both *in vitro* and *in vivo* has allowed the molecular mechanisms of the disease to be investigated further. Common in all types of model is the oxidative stress mechanism leading to a cascade of events and further neuronal damage. Research on these substances may become more widespread if putative studies continue to deliver promising results.

## 5. Herbal Products and Neurodegenerative Diseases

Synthetic drugs cause undesirable adverse effects, whereas natural products are generally considered safe and effective [65,66]. Herbal products have been used throughout history and within every culture to prevent and treat various diseases. Herbal medicines are becoming popular to improve quality of life with either no or limited side effects. The concept of developing antioxidant agents from herbs is much older, yet increasing importance is being given to herbal products that include investigating active constituents of plants and phytomedicines with a view to discover new antioxidant potential therapeutic compounds for treating various neurodegenerative diseases [67]. Although several agents such as vitamins with antioxidant properties have been proved to be beneficial in neurodegenerative diseases, the ability of these vitamins to act as pro-oxidants by inducing oxidative damage and oxidative stress was also reported. Antioxidant vitamins such as tocopherols, ascorbate, retinol and folic acid have been shown to have pro-oxidant effects at higher doses or under certain conditions [68,69]. In a recent study, repetitive administration of vitamin C injection induces dopaminergic neurotoxicity through generation of oxidative stress, and that this toxicity is related to the decline of GSH in both the SNpc and striatum [70]. Further a number of clinical studies on vitamins and antioxidant supplements have shown to have limited or no significant effect on health [71,72]. Majority of these studies and their conclusions are often drawn from experiments conducted with single antioxidant supplement. On the other hand natural herbs are a mixture of antioxidants and thus may work with different kinetics and dynamics. Plant materials including vegetables, fruits, roots, leaves, barks, oilseeds, grains and spices have been screened and used as an alternative therapeutics in preventing various forms of neuronal cell loss, including the nigrostriatal degeneration seen in PD based on their antioxidant properties [73,74,75]. 

## 6. Antioxidant Herbs in Toxin-Induced PD Models

Nerve cell death from oxidative stress has been implicated in a variety of pathologies, including PD. Thus, it is reasonable to use antioxidants to prevent or halt the progression of the disease caused by oxidative stress. In the following sections, the available recent literature on the therapeutic benefits of natural herbs, the utilized parts, the type of extract, and doses used for preventing and/or treating of PD with respect to manipulating ROS levels by scavenging free radicals and targeting redox-sensitive signaling molecules at the signal-transduction, transcription, or death-execution levels in toxin-induced PD models both *in vitro* and *in vivo* are discussed.

### 6.1. Chrysanthemum indicum

*C. indicum* Linn is an herb that belongs to the family Asteraceae and is distributed widely in China, Korea, and other Asian countries. *C. indicum* has been traditionally used since ancient times as a folk and Oriental medicine with immense ethnopharmacological benefits. The tea prepared from the *C. indicum* flower is a popular drink in China [76]. *C. indicum* has been used to treat inflammation, headache, ulcerative colitis, vertigo, eye irritation, hypertension, and respiratory diseases [77,78,79,80]. But, attention is particularly focused on its neuropharmacological actions, including its memory enhancing activity. The effect of a *C. indicum* ethanol extract against MPP^+^-induced damage in SH-SY5Y cells was evaluated by our group. The results indicated that *C. indicum* at 1, 10, and 100 µg inhibited cell loss, decreased ROS production, regulated the Bax/Bcl-2 ratio, and inhibited poly (ADP-ribose) polymerase (PARP) proteolysis in MPP^+^-induced SH-SY5Y cells [80]. Our investigation scientifically supported the long history and safe use of *C. indicum* as an important functional food with potential benefits for ameliorating the neurodegeneration seen in PD. In another study, the antioxidant properties of *C. indicum* ethanol extract were characterized *in vitro* using different methods, including 1, 1-diphenyl-2-picrylhydrazyl, 2,2'-azino-bis(3-ethylbenzothiazoline-6-sulfonic acid) diammonium salt, hydroxyl, superoxide and nitrite radical scavenging, reducing (Fe_3_^+^ to Fe_2_^+^) power, inhibition of linoleic acid oxidation, as well as the prevention of free radical-induced DNA damage [81]. The authors suggested that the *C. indicum* extract was a potent source of natural antioxidants. The protective effects of *C. indicum* might be due to the compounds present in the extract such as flavonoids, terpenoids, and polyphenols, which display various biological activities including antioxidant effects [82,83].

### 6.2. Gastrodia elata

*G. elata* Blume belongs to the family Orchidaceae and has been used as a traditional herbal medicine with numerous therapeutic applications for headaches, dizziness, vertigo, and convulsive illnesses such as epilepsy and tetanus since ancient times [84]. This plant is distributed widely in Nepal, Bhutan, India, Japan, Korea, Siberia, and Taiwan as well as on mainland China. It grows at elevations of 400–3,200 meters at the edge of forests. *G. elata* is effectively used as an anticonvulsant, analgesic, sedative for vertigo, and in patients with general paralysis, epilepsy, and tetanus [85]. *G. elata* inhibits glutamate-induced apoptosis in neurons [86], inhibits neuronal damage in kainite-induced seizure [87], inhibits MPP^+^-induced cytotoxicity in SH-SY5Y cells [88], and protects against neuronal cell damage after transient global brain ischemia [89]. 

In a recent study we investigated the *G. elata* extract and its major bioactive components on MPP^+^-treated MN9D DAergic cells, a classic *in vitro* PD model. The results indicated that vanillyl alcohol, one of the major active biocompounds in *G. elata*, effectively inhibited cytotoxicity and improved cell viability in MPP^+^-induced MN9D DAergic cells. Vanillyl alcohol attenuated the elevation in ROS levels, decreased the Bax/Bcl-2 ratio, and PARP proteolysis. Vanillyl alcohol (1, 10, and 20 µM) protected DAergic MN9D cells against MPP^+^-induced apoptosis by relieving oxidative stress and modulating the apoptotic process [90]. In another study, Shin *et al.* [91] investigated the effects of *G. elata* against methamphetamine (MA)-induced striatal DAergic toxicity in mice. Treatment with MA (7.5 mg/kg, *i.p.* × 4) resulted in significant decreases in behavioral activity, DA level, tyrosine hydroxylase (TH) activity, and TH protein expression. Additionally, MA treatment resulted in significant increases in lipid peroxidation, protein oxidation, and ROS formation. The authors showed that *G. elata* (500 or 1,000 mg/kg, *p.o.*), significantly attenuated MA-induced behavioral and DAergic impairment, and oxidative stress in a dose-dependent manner thereby exhibiting anti-DAergic effects in response to the MA insult *via*, at least in part, inhibiting oxidative stress in the striatum of the mice [91].

### 6.3. Ginseng

Ginseng, the root of *Panax ginseng* C.A. Meyer, belongs to the family Araliaceae and is a valuable herb in traditional Chinese medicine. Ginseng has been utilized for over 2,000 years with the belief that it is a general tonic to promote vitality, health, and longevity. *P. ginseng* roots may be taken orally for diverse benefits, such as an aphrodisiac, a stimulant, to treat type II diabetes, or for sexual dysfunction in men. Ginseng has been included in small doses in energy drinks and teas [92]. It may be found in cosmetic preparations as well, but has not been shown to be clinically effective. A ginseng water extract has been used to treat many kinds of diseases including anemia, insomnia, ischemia, diabetes mellitus, and gastritis [93,94,95,96]. A ginseng extract alleviated scopolamine-induced learning disability and improved spatial working memory in mice [97]. The major effect of ginseng extract resides in its ability to scavenge hydroxyl [98,99] and superoxide radicals [100]. The neurotrophic and neuroprotective effects of ginseng in cell lines and animals have recently been reviewed [96]. Hu *et al.* [101] investigated the protective effects of a water extract of ginseng against MPP^+^-induced cytotoxicity in SH-SY5Y human neuroblastoma cells. The authors suggested that overproduction of ROS, elevated Bax/Bcl-2 ratio, release of cytochrome *c* and activation of caspase-3 expression in MPP^+^-treated SH-SY5Y cells were significantly ameliorated by 0.01, 0.1, and 0.2 mg/mL of a ginseng water extract. An in-depth study revealed that the possible mechanism for the neurotoxic protective effect in SH-SY5Y cells was due to its strong antioxidant properties for suppressing excessive ROS generation [101]. Luo *et al.* [102] studied the protective effect of panaxatriol saponins extracted from *Panax notoginseng* against MPTP-induced neurotoxicity *in vivo*. These authors detected an effect of *P. notoginseng* on MPTP-induced behavioral impairment, and other oxidative parameters in the anatomical region of the substantia nigra pars compacta (SNpc) region of mice brain tissues. They concluded that the protective effects of *P. notoginseng* (100 mg/kg, twice daily for 7 days) might partly be due to its ability to enhance antioxidant capacity and inhibit mitochondrial-mediated apoptosis [102].

### 6.4. Polygala tenuifolia

*Polygalae radix* (PRE) is the root of *P. tenuifolia* Willd and belongs to the Polygalaceae family. *P. tenuifolia* is widely distributed in China and other Asian countries and has been commonly used to treat central nervous system disorders in traditional Korean and Chinese medicine. *P. tenuifolia* is one of the most prescribed herbal remedies for treating various cognitive symptoms associated with aging, senile dementia, and PD [103,104,105]. In a study by Choi *et al.* [106] PRE was investigated for its protective effects and possible mechanisms of action against toxin-induced neuronal death in a mouse model of PD. PC12 cells were damaged by 6-OHDA *in vitro*, and ROS, nitric oxide production and caspase-3 activation were assayed. The authors also evaluated the protective effects of PRE on MPP^+^-induced neurotoxicity in rat primary DAergic neurons and in an *in vivo* mouse model of PD. The results indicated that PRE significantly inhibits 6-OHDA-induced cell damage at doses of 0.05–1 µg/mL with a maximal effect at 0.1 µg/mL. Caspase-3 activity, ROS and NO production were alleviated at 0.1 µg/mL. PRE (0.1 µg/mL) protected mesencephalic DAergic neurons against MPP^+^-induced toxicity and protected DAergic neurons and fibers from MPTP-induced toxicity in the SNpc and striatum *in vivo* when administered orally at 100 mg/kg/day for 3 days. It was concluded that PRE has protective effects on DAergic neurons via its antioxidant and antiapoptotic effects [106]. In another study, the active constituent tenuigenin was studied for its neuroprotective effects in 6-OHDA-induced cytotoxicity in SH-SY5Y cells [107]. These authors indicated that a 10 µM dose of tenuigenin significantly promotes cell viability and reduces cell death. Tenuigenin also protects the mitochondrial membrane potential against 6-OHDA damage and significantly increases glutathione and SOD expression. The authors suggested that the neuroprotection to DAnergic neurons provided by tenuigenin from 6-OHDA-induced damage might partly involve its strong antioxidative effects.

### 6.5. Bacopa monnieri

*B. monnieri Linn*. is in the family Plantaginaceae and a common herb that grows throughout India, Nepal, Sri Lanka, China, Taiwan, Vietnam, as well as in Florida, Hawaii and other southern states of the US where it grows in damp conditions by ponds or in bogs. *B. monnieri* has been used in Indian Ayurvedic traditional medicine for almost 3,000 years and is classified as one of the most important herbs used to improve memory and cognition as well as a potent nerve tonic [108,109]. *B. monnieri* is well tolerated without side effects [109], and the LD50 in rats is as high as 2.7 g/kg when administrated orally [110]. In a study conducted by Singh *et al.* [111], *B. monnieri* protected a DAergic SK-N-SH cell line against MPP^+^- and paraquat-induced toxicity in various survival assays. *B. monnieri* pretreatment (10 mg/mL) significantly attenuated the generation of intracellular ROS and decreased mitochondrial superoxide levels. Furthermore, *B. monnieri* treatment activated the Nrf2 pathway by modulating Keap1 expression, thereby upregulating endogenous GSH synthesis. The authors concluded that *B. monnieri* might be useful in age-related neurodegeneration including PD by preserving cellular redox homeostasis and mitochondrial activities [111]. In an earlier study Hosamani and Muralidhara [112], investigated a standardized *B. monnieri* powder against rotenone-induced oxidative stress and neurotoxicity in *Drosophila melanogaster* exposed to *B. monnieri* powder (0.05 and 0.1%) for 7 days in the diet. The results showed significant attenuation in the levels of endogenous oxidative markers such as malondialdehyde, hydroperoxide, and protein carbonyl content. Complete protection was offered by *B. monnieri* against rotenone (500 mM)-induced oxidative stress and further markedly inhibited DA depletion (head region, 33%; body region, 44%) in the flies. The authors hypothesized that *B. monnieri* may have the ability to mitigate rotenone-induced oxidative stress [112].

In much more recent studies, Shinomol *et al.* tested the hypothesis that a *B. monnieri* extract could offset neurotoxicant-induced oxidative dysfunction in the developing brain of rotenone and 3-nitropropionic acid mouse models [113,114,115,116]. They indicated that rotenone-induced (1.0 mg/kg b.w./day, i.p.) oxidative stress and cell death *in vitro* was attenuated significantly by pretreatment with *B. monnieri* (2, 4, and 6 µg) in DAergic N27 cell lines. *In vivo* studies revealed that *B. monnieri* (5 mg/kg b.w. once daily for 7 days, i.p.) normalized the levels of oxidative markers (malondialdehyde, ROS, and hydroperoxides) and restored depleted GSH levels in rotenone-induced (1 mg/kg b.w. /day for 7 days) mice. *B. monnieri* also effectively normalized protein carbonyl content in all brain regions, suggesting an ability to prevent protein oxidation. Furthermore, oxidative stress and mitochondrial dysfunction in DAergic (N27) cells and prepubertal mouse brain induced by 3-nitropropionic acid were significantly attenuated as evidenced by restoration of the ETC enzyme activities (NADH: ubiquinone oxidoreductase, NADH: cytochrome *c* reductase, succinate-ubiquinone oxidoreductase, and cytochrome *c* oxidase) as well as mitochondrial viability. The activities of antioxidant enzymes (SOD, GPx, glutathione reductase, and thioredoxin reductase), Na (+), K (+)-ATPase, and citric acid cycle enzymes in the striatum that were discernible among 3-NPA mice were restored significantly upon *B. monnieri* pretreatment. In addition, pretreatment with *B. monnieri* for 10 days followed by a 3-NPA challenge (75 mg/kg b.w./day, i.p.) completely prevented 3-NPA-induced oxidative dysfunction in the striatum and other brain regions. Elevated oxidative marker levels (malondialdehyde, ROS, hydroperoxide, and protein carbonyls) were predominantly abolished among mice given *B. monnieri* prophylaxis. The neuroprotective effects of *B. monnieri* may be wholly or in part related to its propensity to scavenge free radicals, maintain redox status, and upregulate antioxidant machinery in striatal mitochondria. The ability of a *B. monnieri* ethanol extract to enhance reduced glutathione and antioxidant defenses in brain regions has been hypothesized for its potent neuroprotective action [116].

### 6.6. Hyoscyamus niger

*H. niger* Linn, commonly known as Henbane in English and Prasikayavani in Sanskrit [117], belongs to the family Solanaceae and is widely distributed in Europe and Asia. This plant contains tropane alkaloids (hyoscyamine, hyoscine, and scopolamine) as well as flavonol glycosides (quercitin, rutin, and kaempferol), which are among the oldest drugs used in medicine [118]. In the traditional Indian medical system, Ayurveda, powdered seeds of *H. niger* are one of the four plant ingredients used for treating PD [119,120]. Senugupta *et al.* (2010) recently investigated the efficacy of *H. niger* for its antiparkinsonian effects in an MPTP model of PD in mice. Parkinsonian mice were treated twice daily with the extract (125–500 mg/kg, p.o.) for 2 days, and motor functions and striatal DA levels were assayed. The *H. niger* extract significantly attenuated motor disability and striatal DA loss in the MPTP-treated mice. The *H. niger* seed extract also significantly inhibited monoamine oxidase activity and attenuated MPP^+^-induced hydroxyl radical generation in isolated mitochondria. The authors reported that the *H. niger* extract showed antiparkinsonian effect by its ability to inhibit increased hydroxyl radicals generated in mitochondria [121]. 

### 6.7. Hibiscus asper

*H. asper,* in the family Malvaceae, is an important medicinal plant widely distributed throughout tropical Africa and Madagascar. A methanol extract of *H. asper* leaves possesses a wide spectrum of biological activities, including *in vitro* antioxidant activity, anxiolytic and antidepressant actions, as well as positive effects on spatial memory formation [122]. Hritcu *et al.* [123] evaluated the relationship between the antioxidant and antiapoptotic actions of a methanol extract of *H. asper* leaves and its neuroprotective properties in a 6-OHDA lesioned rat model of PD. The authors suggested that chronic administration of the methanol extract (50 and 100 mg/kg, i.p., daily for 7 days) significantly increases antioxidant enzyme activities (SOD, GPX and CAT), total GSH content, and reduces lipid peroxidation (MDA level) in rat temporal lobe homogenates, suggesting that antioxidant activity is the major mechanism for its potent neuroprotective effect against 6-OHDA-induced PD in a rat model [123]. In another study, a *H. asper* leaf methanol extract was evaluated against unilateral 6-OHDA lesions in Wister rats. * H. asper* leaves at 50 and 100 mg/kg showed 2,4-dinitrophenyl-1-picryl hydrazyl (DPPH) radical scavenging activity and β-carotene bleaching inhibition activity [122].

### 6.8. Melissa officinalis

*M. officinalis* belongs to the Laminaceae family and is a perennial herb native to southern Europe and the Mediterranean region. Preparations derived from the aerial part of *M. officinalis* are often used in folk medicine to treat various symptoms from the common cold to cardiac dysfunction and ulcers [124]. Data have also supported a protective role for *M. officinalis* intake against AD [125]. Martins *et al.* [126] studied the protective effect of a *M. officinalis* aqueous extract against Mn-induced oxidative stress in chronically exposed mice. Mn is an essential element for biological systems; however, occupational exposure to high levels of this metal may lead to neurodegenerative disorders resembling PD. Mice were administered Mn (50 mg/kg/day) during the first 15 days followed by 100 mg/kg/day for an additional 75 days in drinking water. The experiment was continued with Mn and/or *M. officinalis* treatment for an additional 3 months in drinking water. Mn-treated mice showed a significant increase in TBARS levels (a marker of oxidative stress) in both the hippocampus and striatum. Decreases in total thiol content in the hippocampus and a significant increase in antioxidant enzyme activity (SOD and CAT) in the hippocampus, striatum, cortex, and cerebellum was also observed. Co-treatment with a *M. officinalis* aqueous extract significantly inhibited antioxidant enzyme activity and attenuated the oxidative damage (TBARS and decreased total thiol levels). The authors suggested that the *M. officinalis* aqueous extract possesses potent antioxidative properties, validating its efficacy in attenuating Mn-induced oxidative stress in the mouse brain as seen in PD [126]. 

### 6.9. Cassia obtusifolia

Cassia semen (sickle pod) is the seed of the annual plant *C. obtusifolia* Linn in the Leguminosae family. It is widely cultivated in Korea and China and commonly drunk as a roasted tea and used as a medicinal food. *C. obtusifolia* has multiple therapeutic actions related to the prevention of dementia and ischemia [127]. Ju *et al.* [128] studied the protective effects of *C. obtusifolia* in *in vitro* and *in vivo* PD models. In PC12 cells, *C. obtusifolia* (0.1–10 µg/mL) attenuates cell damage induced by 6-OHDA (100 µM)-induced stress in the MTT assay and inhibits the overproduction of ROS, GSH depletion, mitochondrial membrane depolarization, and caspase-3 activation. In addition, *C. obtusifolia* shows radical scavenging activity in DPPH and 2,2-azinobis-(3-ethyl-benzthiazoline-6-sulphonic acid (ABTS) assays. The authors suggested that *C. obtusifolia* inhibits cell loss against 6-OHDA-induced DA neural toxicity via an antioxidant and antimitochondrial-mediated apoptosis mechanism in PC12 cells [128]. Furthermore, *C. obtusifolia* (0.1–1 µg/mL) extract protected DA cells in a mesencephalic DAergic culture against 6-OHDA- (10 µM) and MPP (10 µM)-induced toxicity. An *in vivo* behavioral test (pole test), revealed that treatment of an MPTP-induced group (30 mg/kg, 5 days) with *C. obtusifolia* (50 mg/kg, 15 days) decreased time to turn and time for locomotion activity, which were longer in the MPTP-alone treated group and significantly protected DA neuronal degeneration in the substantia nigra and striatum in an MPTP-induced mouse model of PD [128]. In another study, an ethanol extract of *C. obtusifolia* seeds was investigated in primary mouse hippocampal cultures exposed to three models of cell death implicated in neurodegeneration such as acute and endpoint studies related to excitotoxicity induced by NMDA, a model of mitochondrial dysfunction induced by incubation with 3-NP, and amyloid-β-protein-induced cell death resulting from incubation with medium from β-amyloid-producing N2a cells. *C. obtusifolia* at 1 and 10 µg/mL was neuroprotective against the mitochondrial toxin 3-NP (1 mM), and Aβ toxicity (8.2 pg/mL), particularly against mitochondrial dysfunction in hippocampal cultures, pointing to a role in the regulation and maintenance of cellular homeostasis and apoptosis, which are relevant for future therapeutic considerations in the treatment of neurodegenerative disorders [129].

### 6.10. Croton celtidifolius

*C. celtidifolius* Baill, in the family Euphorbiaceae, is a tree frequently found in the Atlantic forests of southern Brazil. The *C. celtidifolius* bark has several neurobiological activities including antioxidant and antiinflammatory properties [130,131]. Moreira *et al.* [132] demonstrated that the proantho-cyanidin-rich fraction (PRF) obtained from *C. celtidifolius* bark possesses a wide spectrum of psychopharmacological properties in rats, and that these effects could be attributed to the presence of various catechin and/or proanthocyanidin antioxidant compounds. The same group investigated the effects of *C. celtidifolius* PRF on the neurochemical and behavioral alterations induced by a single intranasal MPTP administration in rats. Pretreatment with PRF (10 mg/kg, i.p.) during 5 consecutive days prevented the inhibition of mitochondrial complex-I in the striatum and olfactory bulb, as well as decreased TH expression in the olfactory bulb and SN of rats infused with MPTP (1 mg/nostril). Moreover, PRF pretreatment attenuated short-term social memory deficits, depressive-like behavior, and reduced locomotor activity observed at different periods after intranasal MPTP administration in rats. The authors indicated that the beneficial effects of PRF in MPTP-intoxicated rats might be due to the presence of various catechin and/or proanthocyanidin compounds, which have profound antioxidant actions [132].

### 6.11. Gynostemma pentaphyllum

*G. pentaphyllum*, a twisting vine orchid in the family Cucurbitaceae, is indigenous to the southern reaches of China, South Korea, and Japan. Gypenosides, which are saponins extracted from this plant, have various bioactivities such as hepatoprotection, antihyperlipidemia, and anticancer effects [133,134,135,136]. This plant is known for its powerful antioxidant and adaptogenic effects purported to increase longevity. Earlier reports indicated that *G. pentaphyllum* protects rat cortical cells against glutamate-induced oxidative injury by preventing GSH depletion and lipid peroxidation [137]. Wang *et al.* [138] explored the neuroprotective effects of *G. pentaphyllum* in an MPTP-induced mouse model of PD. Co-treatment with *G. pentaphyllum* (100, 200, and 400 mg/kg) after acute administration of MPTP (20 mg/kg at 2-h intervals) attenuated the decreased GSH content and reduced SOD activity in the substantia nigra. Furthermore, the loss of nigral DAergic neurons and motor dysfunction, which resulted in oxidative stress, was reversed significantly. The authors suggested that the neuroprotective effect of *G. pentaphyllum* in PD models may be attributed to increased antioxidation capacity [138]. In another study, oral administration of an herbal ethanol extract from *G. pentaphyllum* (10 and 30 mg/kg) starting on day 3 post-lesion with 6-OHDA for 28 days markedly ameliorated the reduction in TH-immunopositive neurons induced by 6-OHDA in the *substantia nigra*. *G. pentaphyllum* administration also recovered the levels of DA, 3,4-dihydroxyphenylacetic acid, homovanillic acid, and norepinephrine in post-lesion striatal tissues. Four gypenoside derivatives such as gynosaponin TN-1, gynosaponin TN-2, gypenoside XLV, and gypenoside LXXIV were identified from *G. pentaphyllum*. The authors suggested that *G. pentaphyllum* might be helpful in the prevention of PD [139].

### 6.12. Thuja orientalis

*T. orientalis* Linn, an evergreen coniferous tree in the family Cupressaceae, is native to North America and eastern Asia. The leaves of *T. orientalis* are commonly used in traditional oriental medicine. They exert an antioxidant effect by scavenging free radicals and have a protective effect on DNA oxidation and red blood cell hemolysis [140]. Earlier reports suggested that the active principle factor, 15-methoxypinusolidic acid (15-MPA), a pinusolide derivative isolated from a *T. orientalis* methanol extract, has neuroprotective effects. 15-MPA protects against glutamate-induced neurotoxicity in primary cultured rat cortical cells by reducing nitric oxide overproduction, GSH depletion, and lipid peroxidation [141,142]. As *T. orientalis* and its compounds suppress oxidative stress, Ju *et al.* [143] investigated the protective effects of standardized *T. orientalis* leaves against 6-OHDA-induced neurotoxicity in SH-SY5Y cells. The results indicated that *T. orientalis* (1000 µg/mL) shows strong radical scavenging effects in 2,2-diphenyl-2-picrylhydrazyl and 2,2-azinobis-(3-ethylbenzthiazoline-6-sulphonic acid) assays. Furthermore, *T. orientalis* (10 µg/mL) reduces intracellular ROS production induced by 6-OHDA (200 µg/mL). Additionally, *T. orientalis* blocks the reduction in mitochondrial membrane potential, the release of cytochrome *c*, and caspase-3 activation. Moreover, *T. orientalis* decreases the phosphorylation of extracellular signal-regulated kinase, which has pro-apoptotic functions. The authors indicated that the protective effects of *T. orientalis* in SH-SY5Y cells induced by 6-OHDA might partly be through downregulation of oxidative stress. 

### 6.13. Paullinia cupana

*P. cupana* Mart. var. Sorbilis is a Brazilian plant in the Sapindaceae family. The chemical composition of *P. cupana* seeds is quite complex and includes catechin, epicatechin, ent-epicatechin, procyanidins, and phenolic compounds [144]. Some of these components have nigrostriatal DAergic neuroprotective and antioxidant activities [138,145]. The presence of xanthine bases such as caffeine, theophylline, and theobromine, as well as purine alkaloids, which have been described as having antioxidant properties, is also well known [146,147,148,149]. The antioxidant activity of a *P. cupana* ethanol extract has been reported [150]. In a recent study de Oliveira *et al.* [151] investigated the effects of commercial powdered *P. cupana* seeds against rotenone-induced cytotoxicity in SH-SY5Y cells as an *in vitro* model of DAergic neuron degeneration. The *P. cupana* extract (0.312 and 0.625 mg/mL) significantly and dose-dependently increased viability of SH-SY5Y cells treated with rotenone (300 nM). Furthermore, the authors also measured lactate dehydrogenase (LDH) levels and analyzed nuclear integrity with Hoechst 33258 stain. LDH levels decreased significantly by adding 0.312 mg/mL of *P. cupana*, but, unexpectedly, no changes were observed at the higher concentration. Moreover, chromatin condensation and nuclear fragmentation decreased significantly by adding both extract concentrations. The authors suggested that the compounds with potential antioxidant activity present in *P. cupana* might be responsible for such profound protective action against rotenone toxicity in SH-SY5Y cells [151]. 

### 6.14. Morus alba

Mulberry (*M. alba* L.) is a member of the Moraceae family and is native to northern China. This plant has been naturalized and is cultivated widely everywhere. Mulberry fruit is commonly eaten, often dried, or made into wine. In traditional oriental medicine, it has been used to treat premature grey hair, to nourish the blood, to treat constipation and diabetes and to generate body fluids, which generally means enhancing health and promoting longevity [152]. In addition, it also ameliorates inflammation-related hematological parameters in carrageenan-induced arthritic rats [153], promotes recovery from physical stress [154], has neuroprotective effects against cerebral ischemia [155]. and has radical-scavenging properties [156]. Mulberry fruit contains not only high amounts of anthocyanins [157,158], a subset of the flavonoids that are important natural antioxidants [159,160], but also nonanthocyanin phenolics including rutin and quercetin that have multi-bioactive functions including neuroprotective effects [155,157,158,161]. Kim *et al.* [162] investigated the protective effects of mulberry fruit (ME) in a toxin-induced PD model. A 70% ethanol extract of ME (1, 10 and 100 µg/mL) significantly protected SH-SY5Y cells from neurotoxicity induced by 150 µM 6-OHDA in a dose-dependent manner. The protective effect of ME was mediated by its antioxidant and antiapoptotic effects, by regulating ROS and NO generation, Bcl-2 and Bax proteins, mitochondrial membrane depolarization, and caspase-3 activation. Furthermore, ME protected mesencephalic primary cells stressed with 6-OHDA (10 µM) or MPP^+^ (10 µM). In an *in vivo* subacute mouse PD model induced by MPTP (30 mg/kg), ME at 500 mg/mL showed a preventative effect against PD-like symptoms (bradykinesia) in a behavioral test and prevented MPTP-induced DAnergic neuronal damage in an immunocytochemical analysis of the SNpc and ST. The authors concluded that the mulberry fruit extract significantly protected neurons against neurotoxins in *in vitro* and *in vivo* PD models through its antioxidant and anti-apoptotic effects [162]. The antioxidant activity of teas essences prepared from mulberry, *Camellia sinensis L*., and *Cudrania tricuspidata* (Carr.) Burea, have been examined using two antioxidant assays. Selected volatile chemicals identified in these plants were also tested for antioxidant activity. All extracts exhibited antioxidant activity with a clear dose response in aldehyde/carboxylic acid and malonaldehyde/gas chromatography assays [163].

### 6.15. Ginkgo biloba

EGb761 (Figure 5E) is a well-known plant extract obtained from leaves of the *G. biloba*. This patented extract contains two main groups of active compounds [flavonoids (24%) and terpenoids (6%; known as ginkgolides A, B, C, M, J, and bilobalide)]. EGb761 is a potent antioxidant and free radical scavenger. A study evaluated the neuroprotective effect of EGb761 against oxidative stress induced by MPTP in C57BL/6J mice. MPTP administration resulted in a significant decrease in striatal DA levels and TH immunostaining in the striatum and SNpc, and mice that received EGb761 (40 mg/kg) had significantly attenuated MPTP-induced loss of striatal DA levels and TH immunostaining in the striatum and SNpc. The neuroprotective effect of EGb761 against MPTP neurotoxicity is associated with blockade of lipid peroxidation and reduction of superoxide radical production, both of which are oxidative stress indices. The presence of elevated lipid peroxidation was detected 18 days after the last injection of MPTP. EGb761 blocked lipid peroxidation in the MPTP-treated groups. Furthermore, EGb761 regulates other antioxidant enzymes including GPx and glutathione reductase and shows potent inhibitory activity against SOD. Moreover, EGb761 improves MPTP-induced impairment of locomotion in a manner that correlates with enhanced striatal DA levels in behavioral experiments. These findings suggest that EGb761 attenuates MPTP-induced neurodegeneration of the nigrostriatal pathway in mice and that the neuroprotective effects are exerted via antioxidant or free radical scavenging action [164]. 

Several other natural products of plant origin such as a *Uncaria rhynchophylla* Hook extract [165], an isoflavone mixture from *Trifolium pretense* [166], salvianolic acid B isolated from *Salvia miltiorrhiza* [167], 6,7-di-*O*-glucopyranosyl-esculetin extracted from *Fraxinus sieboldiana* [168], baicalein, a flavonoid, obtained from *Scutellaria baicalensis* [169], a leaf extract of *Withania somnifera* [170], fustin, a flavonoid from *Rhus verniciflua* [171,172], *Acanthopanacis senticosus* [173], a rhizome of *Cyperus rotundus* [174], an ethanol extract of *Delphinium denudatum* [175], and a hydroalcohol extract of *Ilex paraguariensis* [176] have protective effects against PD in cellular and animal models based on their antioxidative abilities. A summary of the natural plant antioxidant products showing beneficial effects in *in vitro* and *in vivo* PD models is illustrated in Table 1. 

**Table 1:** Summary of recent antioxidant herbal products reported to be neuroprotective in experimental models of Parkinson’s disease.

Plant name/species	Extract/ constituent	Dose	Toxin/tested subjects	Biological activity and targets	Ref.
*Chrysanthemum indicum*	Ethanol extract	1, 10 and 100 µg	MPP^+^/SH-SY5Y cells	Regulation of Bax/Bcl-2 ratio & decreasing, inhibition of free radicals and oxidative stress	[80] [43]
*Gastrodia elata*	Vanillyl alcohol	1, 10 and 20 µM	MPP^+^/MN9D cells	Regulation of Bax/Bcl-2 ratio & decreasing oxidative stress	[90]
	Methanol extract	500 or 1,000 mg/kg	Methamphetamine/C57BL/6J mice	Decrease oxidative stress	[91]
Ginseng	Aqueous extract	0.01, 01 and 0.2 mg/mL	MPP^+^/SH-SY5Y cells	Decrease in Cytochrome c, Caspace-3 activation & ROS generation	[101]
	Panaxatriol saponins	100 mg/kg, twice daily for 7 days	MPTP/Kunming mice	Improvement in behavioral impairments caused by MPTP	[102]
*Polygala tenuifolia*	Aqueous extract	0.05-µg/mL and 100 mg/kg	6-OHDA/PC12 cells and MPP^+^/ C57BL/6 mice	Decrease in ROS & protection of mesencephalic dopaminergic neurons	[106]
	Tenuigenin	10 µM	6-OHDA/SH-SY5Y cells	Increased expression of glutathione (GSH) and superoxide dismutase (SOD)	[107]
*Bacopa monnieri*	Standardized aqueous extract	10 mg/mL	MPP^+^, paraquat/ SK-N-SH cells	Decrease in intracellular ROS & upregulation of Nrf2	[111]
	Standardized aqueous extract	0.05 and 0.1%	Rotenone/Drosophila melanogaster flies	Ablation of oxidative stress and dopamine depletion	[112]
	Ethanolic extract	2, 4 and 6 µg/1 mg/kg b.w./day, i.p.	Rotenone/N27 cell and prepubertal mice	Decrease oxidative stress & increase Anti-oxidant defense	[116]
	Ethanolic extract	0.5 and 1.0 µg	3-nitropropionicAcid/prepubertal mice	Scavenge free radicals, maintain redox status, and upregulate antioxidant machinery.	[115]
*Hyoscyamus niger*	Seed extract	125–500 mg/kg, p.o.	MPTP/Male Balb/c mice	Alleviation of motor deficits produced by MPTP and inhibition of hydroxyl radical	[1211]
*Hibiscus asper*	Methanolic extract	50 and 100 mg/kg, i.p., daily, for 7 days	6-HDA/Wistar rats	Increased antioxidant defense system like GSH and SOD & decreased MDA levels	[123]
*Melissa officinalis*	Aqueous extract	100 mg/kg/day in the drinking water	Manganeese/ Male albino mice	Decrease in thiobarbituric acid & Increased SOD and catalase	[126]
*Cassia obtusifolia*	Seed extract	0.1-10 µg/mL/50 mg/kg, 15 days	6-OHA/PC12 cells and MPTP/Male C57BL/6 mice	Inhibition of GSH depletioncaspase-3 activation/Decreased T-turn and T-LA time in pole test	[128]
	Seed extract	1.0 and 10 µg	NMDA, 3-NP, Amyloid β/ Hippocampal cultures from C57Bl/6 mice	Reduce excitotoxicity, mitochondrial dysfunction, and Aβ toxicity. regulation and maintenance of cellular homeostasis and apoptosis	[129]
*Croton celtidifolius*	Bark	10 mg/kg, i.p.	MPTP/Wistar rats	Prevention of mitochondrial complex-I inhibition & improvement in memory deficits	[132]
*Gynostemma pentaphyllum*	Gypenosides	100, 200 and 400 mg/kg	MPTP/C57BL/6 mice	Increase in SOD and GSH content & survival of nigral dopaminergic neurons	[177]
*Thuja orientalis*	Ethanolic leaf extract	10 µg/mL	6-OHDA/SH-SY5Y cells	Decrease in ROS production	[143]
*Paullinia cupana*	Seed powder	0.312 and 0.625 mg/mL	Rotenone/SH-SY5Y cells	Survival of SH-SY5Y cells exposed to rotenone	[151]
Mulberry	Ethanol extract	1, 10 and 100 µg/mL/500 mg/mL	6-OHDA/SH-SY5Y cells and MPP^+^/Male C57BL/6 mice	Decrease in ROS, NO & Caspase-3 generation/Improvement in behavioral deficits	[162]
*Ginkgo biloba*	EGb761 extract	40 mg/kg	MPTP/C57BL/ 6J mice	Decrease in lipid peroxidation, increase in striatal dopamine and locomotor activity	[164]
*Uncaria rhynchophylla*	Aqueous extract	0.1, 0.5 and 1.0 µg ( *in vitro*) and 5 mg/kg/day for 14 days (*in vivo*)	6-OHDA/PC12 cells and6-OHDA/ Sprague–Dawley rats	Reduced cell death, generation of ROS, increased GSH levels, and inhibited caspase-3 activity. Lowered dopaminergic cell loss, antioxidative and anti-apoptotic activities.	[165]
*Trifolium pretense*	Aqueous extract	0.5, 1 and 2 µg/mL	hydrogen peroxide/HCN 1-A cells	Antioxidant activity	[166]
*Salvia miltiorrhiza*	Salvianolic acid B	0.1, 1.0 and 10 µM	6-OHDA/SH-SY5Y cells	Redox regulation and antioxidant effect	[167]
*Hypericum perforatum*	Flavonoid rich extract	3.125, 12.5 and 25 µg/mL	hydrogen peroxide/PC12 cells	Elevated the cell viability decreased the levels of LDH release and decreased the occurrence of apoptotic cells. Antioxidant and apoptotic activity	[178]
*Fraxinus sieboldiana*	6,7-di- *O*-glucopyranosyl-esculetin	0.1, 1 and 10 µM	Dopamine/SH-SY5Y cells	Redox regulation and antioxidant properties	[179]
*Scutellaria baicalensis*	Baicalein	0.05, 0.5 and 5 µg/mL	6-OHDA/SH-SY5Y cells and Sprague–Dawley rats	Antioxidant and antiapoptotic activities	[169]
*Withania somnifera*	Aqueous root extract	100 mg/kg	MPTP/ C57BL/6 mice	Regulation of redox status and antioxidant effects	[170]
*Cyperus rotundus*	Aqueous rhizome extract	50 and 100 µg /mL	6-OHDA/PC12 cells	Antioxidant and antiapoptotic activities	[174]
*Delphinium denudatum*	ethanolic extract	200, 400 and 600 mg/kg	6-OHDA/Rats	Regulation of antioxidative enzymes and antioxidant effects	[175]
Ilex paraguariensis	Hydro-alcoholic extract	250 and 500 mg/kg	MPTP/C57BL/6 mice	Antioxidant activity	[176]

## 7. Conclusions and Future Perspectives

This study searched the literature for the most recent available data about antioxidant plants/constituents that possess neuroprotective activity in experimental models of PD. Despite the great variety of plants, only a few have had their pharmacological effects investigated for antiparkinsonian activity; thus, there are huge prospects in this field for future studies with plants and their bioactive molecules. Notably, the plants discussed in this review have played important roles for centuries as pharmacologically efficient therapeutics against brain diseases. These medications have been validated by traditional use and are time tested, as compared to modern day supplements. Growing evidence suggests a model for redox homeostasis in which the ROS-antioxidant interactions act as a metabolic interface for signals derived from metabolism and the environment. Several redox regulating transcriptional signaling factors and enzymes are involved during the oxidative stress response. Modulation of these factors by antioxidative herbal products provides important opportunities for neuroprotection based on molecular targeting. 

Synthetic antioxidants are adding up their negative or no effects and are recently highlighted to be less beneficial to human health. Thus the search for effective, nontoxic natural molecules with immense antioxidative activity has been intensified. Antioxidants provide essential information on cellular redox state and they influence gene expression associated with oxidative stress responses to maximize defense. Several molecules have been identified to be responsible in regulating redox status and oxidative stress which is one of the key factors in the etiopathogenesis of neurodegenerative diseases including PD. In addition to endogenous antioxidant defense systems, a promising strategy for targeting redox status of cells is to use readily available natural substances from vegetables, fruits and herbs. Therefore, consumption of plant-derived antioxidants may appear to be a suitable alternative. 

Mounting evidence in randomized control trials indicated that purified antioxidants have produced inconsistent results suggesting that supplementation by natural antioxidant herbs may modulate neurodegeneration. The behavior and bioavailability of the pure antioxidant may differ from that of the same molecule consumed as part of a complex herb matrix [180]. Evidence showed that antioxidant treatment of the natural herbs discussed in the present review results in neuroprotection in experimental models of PD. These herbs can also be utilized as adjuvant therapy with combination of main therapy to ameliorate the symptoms of PD. Moreover, this combination will reduce the side effects and will provide an alternative approach for treatment of PD [181]. The potential clinical benefits deriving from these natural antioxidant herbs is still under wide debate. Further research on their ability to delay or forestall disease progression by protecting or rescuing the DAnergic neurons or even restoring those that have been lost is required.

Future perspectives may include a logical combination of ROS-modulating herbs and their derived molecules that affect redox-sensitive signaling pathways which may further enhance the therapeutic activity when compared to the currently existing treatment regimen. Although the repair of damaged DAergic neurons seen in PD may be beyond the possibilities of natural antioxidants, combinations of antioxidant herbs and supplementation with current drugs may serve a neuroprotective purpose and decrease the risk of condition and progression in PD. Integration of pharmacology, natural product chemistry, medicinal chemistry, clinical and other related disciplines could be the most promising strategy for drug discovery and increase success rate. Studies on improved quality control techniques, authenticity, standardization of the natural herbal molecules and their ability to cross the blood-brain barrier to reach the target sites is quite necessary. Establishing the characteristic chemical “fingerprints” for herbs and herbal products are also warranted.
